# Dock5 Deficiency Promotes Proteinuric Kidney Diseases via Modulating Podocyte Lipid Metabolism

**DOI:** 10.1002/advs.202306365

**Published:** 2023-12-31

**Authors:** Hua Qu, Xiufei Liu, Jiaran Zhu, Xin Xiong, Lu Li, Qingshan He, Yuren Wang, Guojun Yang, Linlin Zhang, Qingwu Yang, Gang Luo, Yi Zheng, Hongting Zheng

**Affiliations:** ^1^ Department of Endocrinology Translational Research of Diabetes Key Laboratory of Chongqing Education Commission of China the Second Affiliated Hospital of Army Medical University Chongqing 400037 China; ^2^ Department of Clinical Laboratory the Second Affiliated Hospital of Army Medical University Chongqing 400037 China; ^3^ Department of Neurology the Second Affiliated Hospital of Army Medical University Chongqing 400037 China; ^4^ Department of Orthopedics the Second Affiliated Hospital of Army Medical University Chongqing 400037 China

**Keywords:** Dock5, lipid metabolism, podocytes, proteinuric kidney diseases

## Abstract

Podocytes are particularly sensitive to lipid accumulation, which has recently emerged as a crucial pathological process in the progression of proteinuric kidney diseases like diabetic kidney disease and focal segmental glomerulosclerosis. However, the underlying mechanism remains unclear. Here, podocytes predominantly expressed protein dedicator of cytokinesis 5 (Dock5) is screened to be critically related to podocyte lipid lipotoxicity. Its expression is reduced in both proteinuric kidney disease patients and mouse models. Podocyte‐specific deficiency of Dock5 exacerbated podocyte injury and glomeruli pathology in proteinuric kidney disease, which is mainly through modulating fatty acid uptake by the liver X receptor α  (LXRα)/scavenger receptor class B (CD36) signaling pathway. Specifically, Dock5 deficiency enhanced CD36‐mediated fatty acid uptake of podocytes via upregulating LXRα in an m^6^A‐dependent way. Moreover, the rescue of Dock5 expression ameliorated podocyte injury and proteinuric kidney disease. Thus, the findings suggest that Dock5 deficiency is a critical contributor to podocyte lipotoxicity and may serve as a promising therapeutic target in proteinuric kidney diseases.

## Graphical abstract

Podocyte‐specific deficiency of Dock5 exacerbated podocyte injury and glomeruli pathology in proteinuric kidney disease. Mechanically, Dock5 deficiency enhanced CD36‐mediated fatty acid uptake of podocytes via upregulating LXRα in an m^6^A‐dependent way. Moreover, rescue of Dock5 expression ameliorated podocyte injury and proteinuric kidney disease. Current study suggests that Dock5 may serve as a promising therapeutic target in proteinuric kidney diseases.

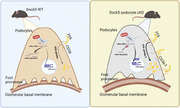

## 1 Introduction

Proteinuric kidney diseases, such as diabetic kidney disease (DKD) and focal segmental glomerulosclerosis (FSGS), are a group of disorders characterized by the presence of excess proteins in the urine. They account for ≈80% of all cases that progress to end‐stage kidney disease (ESRD), ultimately requiring lifelong renal replacement therapy.^[^ ^1^, ^2^ ^]^ Although several therapeutic strategies, including renin‐angiotensin‐aldosterone system (RAAS) inhibitors and lifestyle modifications, have been applied clinically for decades, the prevalence of proteinuric kidney diseases continues to increase worldwide.^[^ ^3^, ^4^ ^]^

Although damage to any component of the glomerular filtration barrier can contribute to proteinuria, extensive studies have shown that podocyte injury plays a fundamental role in the pathogenesis of proteinuric kidney diseases.^[^ ^1^, ^5^ ^]^ Podocytes are specialized terminally differentiated cells that cover the outer surface of the glomerular tuft; therefore, they are vulnerable to a variety of injurious stimuli and are difficult to repair and regenerate after damage. Lipids are essential for maintaining podocyte biological functions, such as serving as energy sources for adenosine triphosphate generation, structural components of biological membranes, and signal transduction molecules.^[^ ^6^ ^]^ Generally, lipid metabolism is well organized, and quite a few lipids accumulate in the podocytes. In proteinuric kidney diseases, excessive lipid accumulation can be found in podocytes, and recent emerging evidence suggests that lipid overload in podocytes may serve as a crucial pathological process in proteinuric kidney diseases, which was defined as podocyte lipotoxicity.^[^ ^7^, ^8^, ^9^ ^]^ Thus, understanding the mechanisms of podocyte lipotoxicity is important for elucidating the etiology and progression of proteinuric kidney disease, and for developing specific treatment strategies.

Dedicator of cytokinesis 5 (Dock5) belongs to the Dock family, a class of guanine nucleotide exchange factors, and is known to be involved in osteoclast function,^[^ ^10^, ^11^ ^]^ actin cytoskeleton,^[^ ^12^, ^13^ ^]^ mast cell degranulation,^[^ ^14^ ^]^ epithelial invasion and metastasis.^[^ ^15^, ^16^ ^]^ Our previous studies have also suggested that it plays a role in wound healing^[^ ^17^ ^]^ and hepatic insulin resistance.^[^ ^18^ ^]^ Here, we showed that Dock5 may be involved in podocyte lipid metabolism by using sodium‐glucose cotransporter‐2 inhibitors (SGLT2i), which were recently reported to ameliorate podocyte lipotoxicity.^[^ ^19^ ^]^ We revealed that Dock5 was predominantly expressed in podocytes of the kidney, and its expression was reduced in both proteinuric kidney disease patients and mouse models. Podocyte‐specific deficiency of Dock5 exacerbated podocyte injury and glomeruli pathology in proteinuric kidney disease, which was mainly through modulating fatty acid uptake through the signaling pathway. Specifically, Dock5 deficiency enhanced CD36‐mediated fatty acid uptake of podocytes via upregulating LXRα in an N6‐methyladenosine (m^6^A)‐dependent way. Moreover, the rescue of Dock5 expression ameliorated podocyte injury and proteinuric kidney disease. Thus, our findings suggest that Dock5 deficiency might be a critical contributor to podocyte lipotoxicity and may serve as a promising therapeutic target in proteinuric kidney diseases.

## 2 Result

### 2.1 Dock5 is Podocytes Predominantly Expressed in the Kidneys and May Link to Podocyte Lipid Metabolism

**Figure 1:**
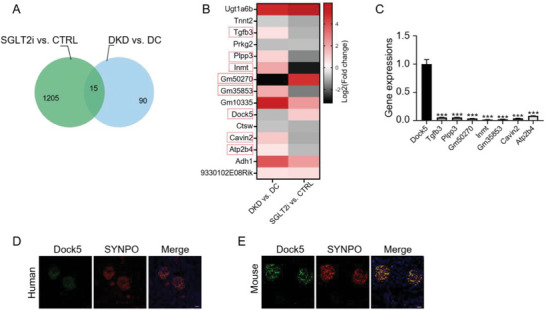
Dock5 is podocytes predominantly expressed in the kidney and may link to podocyte lipid metabolism. A) The Venn diagram showed the overlapped genes between differentially expressed gene (DEG) datasets of podocytes treated by sodium‐glucose cotransporter‐2 inhibitors (SGLT2i), which was recently been reported to ameliorate podocyte lipotoxicity, and podocytes isolated from DKD mice. B) The overlapped DEGs were shown by heatmap. C) mRNA expressions of the indicated gene. D,E) Representative immunofluorescence (IF) images of Dock5 (green) and synaptopodin (SYNPO, red) in renal tissues from healthy humans D) and C57BL/6J mouse E). Data are mean ± SEM. Scale bar: 50 µm in D, 20 µm in E. *n* = 3 for A–C, *n* = 7 subjects for D, *n* = 6 mice for E. Statistical analysis was performed using *t*‐test for C. ^***^ *p* < 0.001 compared with Dock5.

To screen for genes that may be involved in podocyte lipid metabolism, we analyzed the differential gene expression (DGE) dataset of podocytes treated with SGLT2i (with a cut‐off of the fold‐change set at ≥1.5 or ≤0.67, and *p* < 0.05, Table S1, Supporting Information), which was recently reported to ameliorate podocyte lipotoxicity.^[^ ^19^ ^]^ We also analyzed the DGE dataset of podocytes under proteinuric kidney disease by isolating podocytes from DKD mice (with a cut‐off of the fold‐change set at ≥1.5 or ≤0.67, and *p* < 0.05, Table S2, Supporting Information). We then overlapped the two datasets and identified 15 convergence genes. Among them, eight genes exhibited opposite changes between the disease model and SGLT2i treatment (**Figure** **1**A,B). Quantitative reverse transcription polymerase chain reaction (qRT‐PCR) analysis revealed that Dock5 showed markedly higher expression in podocytes than the other seven genes (Figure 1C). Moreover, we assessed the expression pattern of Dock5 in the kidney by querying published single‐cell RNA‐sequencing datasets. As shown in Figure S1 (Supporting Information), Dock5 showed a predominant expression pattern in podocytes of healthy mouse kidneys (Figure S1, Supporting Information).^[^ ^20^ ^]^ This was further confirmed in podocytes from both human and mouse kidney tissues by colocalization analysis of Dock5 with synaptopodin (SYNPO), the known podocyte marker^[^ ^21^ ^]^ (Figure 1D,E).

### 2.2 Dock5 Expression is Reduced in Podocytes From Proteinuric Kidney Disease Patients and Mouse Models

**Figure 2:**
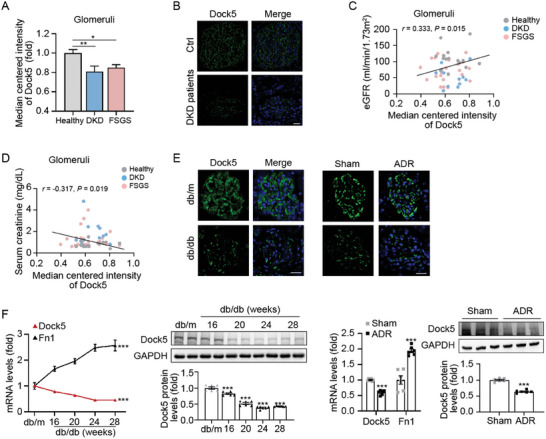
Dock5 shows lower expression in proteinuric kidney disease patients and mouse models. A) Glomerular Dock5 expressions in patients with DKD and FSGS in the Nephroseq database. B) Representative IF images of Dock5 (green) in glomeruli from patients with DKD. C,D) Correlations between glomerular Dock5 expressions and estimated glomerular filtration rate (eGFR, C), or serum creatinine D) in the Nephroseq database. E) Representative IF images of Dock5 (green) in glomeruli from db/db‐induced DKD and ADR‐induced FSGS mice. F) qRT‐PCR and western blot analysis, and their quantification of Dock5 expression in isolated glomeruli from db/db DKD and ADR‐induced FSGS mice. Data are mean ± SEM. Scale bar: 20 µm in B and E. *n* = 12–25 subjects for A, C, and D, *n* = 6–7 subjects per group for B, *n* = 6 mice per group for E and F. Statistical analysis was performed using one‐way or two‐way ANOVA test for A and db/db part of F, *t*‐test for ADR part of F, Spearman's correlations for C and D. ^*^ *p* < 0.05, ^**^ *p* < 0.01, ^***^ *p* < 0.001.

Next, to investigate the possible association between Dock5 and proteinuric kidney diseases, Dock5 expression was observed in patients (DKD and FSGS) and mouse models (db/db‐induced DKD mice and ADR‐induced FSGS mice). By querying the published datasets on kidney disease compiled in the Nephroseq database (nephroseq.org), we found that Dock5 expression was significantly lower in patients with DKD and FSGS than in healthy living donors (**Figure** **2**A). Moreover, we assessed the expression of Dock5 in DKD patients from our hospital and found the same trend (Figure 2B). In addition, Dock5 expression was positively associated with the estimated glomerular filtration rate (eGFR), and negatively correlated with serum creatinine levels, according to Nephroseq (Figure 2C,D). We also detected Dock5 expression characteristics in mouse models using immunostaining, qRT‐PCR, and western blot analyses. Consistent with the observations in patients, Dock5 expression was decreased in both DKD (db/db) and FSGS (ADR) mice as well (Figure 2E,F). Notably, along with the progression of kidney injury (represented by fibronectin 1 (Fn1) expression in Figure 2F), Dock5 showed a gradual downward trend (Figure 2F, left). Taken together, the down‐regulation of Dock5 in patients and mouse models indicates the potential involvement of Dock5 in the progression of proteinuric kidney diseases.

### 2.3 Podocyte‐Specific Deletion of Dock5 Accelerates Podocyte Injury and Glomeruli Pathology in Proteinuric Kidney Diseases

**Figure 3:**
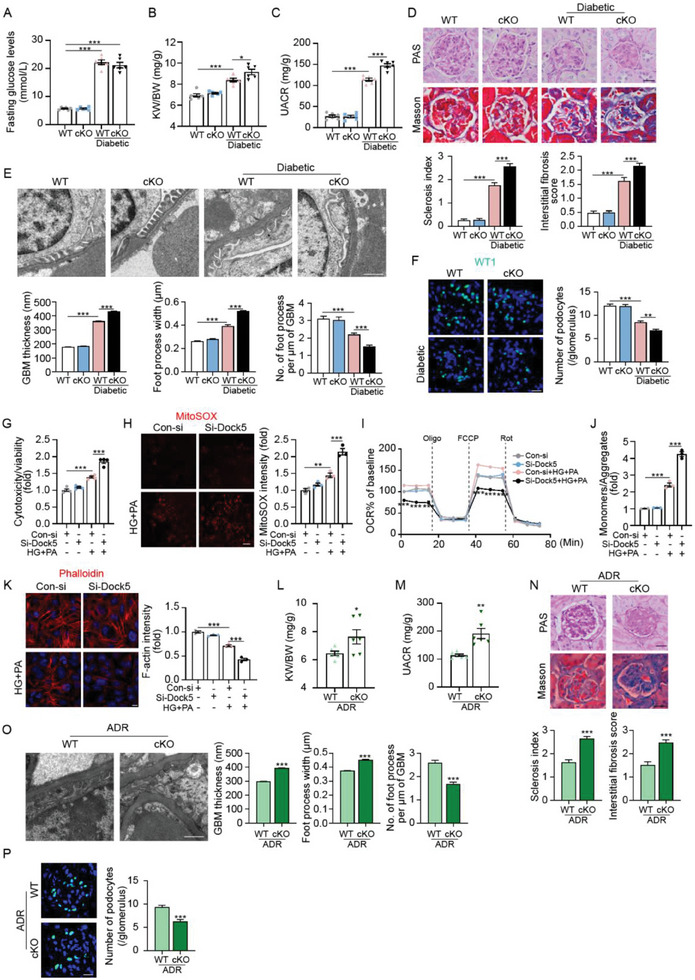
Dock5 deletion in podocytes exacerbates renal injury in proteinuric kidney disease. A–C) Dock5 cKO and its wildtype (WT) littermates induced diabetes by HFD feeding combined with STZ injection. Fasting glucose levels A), kidney weight‐to‐body weight ratio (KW/BW, B), and urine albumin‐to‐creatinine ratio (UACR, C) were assessed. D) Histological changes of glomeruli were evaluated by Periodic acid–Schiff (PAS) and Masson's trichrome (Masson) and were quantified in non‐diabetic and diabetic cKO and WT mice. E) Representative transmission electron microscopy (TEM) images and quantifications of glomerular basement membrane (GBM) thickness, foot process width, and the number of foot processes in mice from indicated groups. F) Representative IF images and quantifications of Wilms’ Tumer 1 (WT1, green) in glomeruli from cKO and WT mice with or without diabetes. G) Cytotoxicity normalized to viability was detected in podocytes treated by Dock5‐specific siRNA combined with or without high glucose and palmitic acid (HG+PA). H–J) Mitochondrial ROS production H), mitochondrial respiration I), and mitochondrial membrane potential J) were assessed by MitoSOX staining, OCR before and after sequential injection of oligomycin (ATP synthase inhibitor), FCCP (protonophoric uncoupler), and rotenone (electron transport inhibitors), and by JC‐1 dye respectively in podocytes treated by si‐Dock5 with or without HG+PA. K) Representative IF images and quantifications of F‐actin by phalloidine staining in podocytes treated by si‐Dock5 with or without HG+PA. L–P) Dock5 cKO and WT mice were induced FSGS by ADR injection. KW/BW L), UACR M), histological changes of glomeruli and quantifications N), representative TEM images and quantifications O), representative IF images and quantifications of WT1 (green) in glomeruli P) were assessed. Data are mean ± SEM. Scale bar: 20 µm in D, F, H, N, and P, 1 µm in E and O, 10 µm in K. *n* = 6 mice per group for A–F and L–P, *n* = 4 for G, *n* = 3 for H–K, *n* = 78–103 glomeruli for D and N, *n* = 10 images per mouse for E and O, *n* = 46–65 glomeruli for F and P. Statistical analysis was performed using one‐way or two‐way ANOVA test for A–K, *t*‐test for L–P. ^*^ *p* < 0.05, ^**^ *p* < 0.01, ^***^ *p* < 0.001.

Then, we investigated the effects of Dock5 downregulation. The podocyte‐specific Dock5 knockout mice (Dock5^fl/fl^‐Cre^+^, cKO) were generated by breeding mice with the Dock5 conditional allele (Dock5^fl/fl^) and those with podocin‐Cre. The knockout models were confirmed by tail genotyping, qRT‐PCR, western blot analyses, and co‐immunostaining with the podocyte marker SYNPO (Figure S2A–C, Supporting Information). The results showed that despite no differences being observed after Dock5 knockdown under basal conditions, cKO mice exhibited a higher kidney weight‐to‐body weight ratio (KW/BW) and urine albumin‐to‐creatinine ratio (UACR) than their wild‐type (WT) littermates under DKD conditions, despite unchanged blood glucose levels (**Figure** **3**A–C). Histological evaluation, including periodic acid‐Schiff (PAS) and Masson's trichrome (Masson) staining, showed more severe glomerular pathology in DKD cKO mice than in DKD WT mice (Figure 3D). Moreover, transmission electron microscopy (TEM) revealed aggravated thickening of the glomerular basement membrane (GBM), podocyte foot process broadening, and effacement in DKD cKO mice (Figure 3E). Exacerbated podocyte loss was also observed by Wilms tumor‐1 (WT1)^[^ ^22^ ^]^ staining (Figure 3F). Consistent with our observations in vivo, enhanced cytotoxicity, mitochondrial dysfunction, and actin cytoskeleton remodeling were observed in a podocyte cell line (MCP5) treated with Dock5‐siRNA under high glucose and palmitic acid (HG and PA) in vitro (Figure 3G–K).

We further observed the effects of Dock5 deficiency on FSGS. Similar to DKD, Dock5 cKO mice under FSGS conditions (ADR‐treated) showed higher KW/BW and UACR, exacerbated glomerular pathology, aggravated thickening of GBM, podocyte foot process broadening and effacement, and podocyte loss (Figure 3L–P). Collectively, these data suggested that Dock5 deficiency exacerbates podocyte injury and glomerular pathology during the progression of proteinuric kidney diseases.

### 2.4 Loss of Dock5 Induces Podocyte Injury Mainly Through Promoting Cd36‐Mediated Fatty Acid Uptake

**Figure 4:**
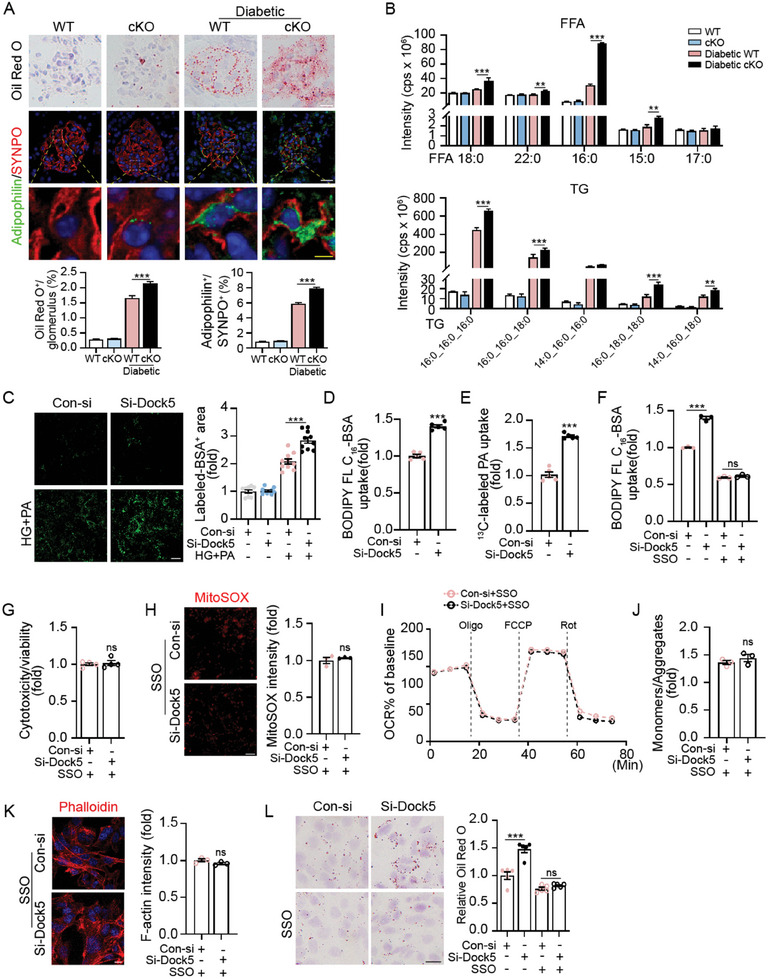
Dock5 deficiency induces podocyte injury mainly by promoting CD36‐mediated fatty acid uptake. A) Representative Oil red O staining and IF images showing lipid accumulation in glomeruli and podocytes (co‐staining adipophilin (green) with SYNPO (red)), and their quantifications were shown below. B) Lipidomics of podocytes isolated from indicated groups were assessed by UPLC‐MS/MS analysis. Ion chromatograms and quantifications of indicated lipid species were shown. C) Representative images and corresponding quantifications of uptake of PA with Alexa Fluor 488‐labeled BSA in podocytes treated by si‐Dock5 with or without HG+PA. D) The uptake of BODIPY FL C_16_‐BSA was quantified by flow cytometry in podocytes treated with si‐Dock5 with HG+PA. E) Lipid uptake was evaluated by ^13C‐labeled^ PA in podocytes treated by si‐Dock5 with HG+PA. F–L) Podocytes were treated by si‐Dock5 and CD36 inhibitor sulfosuccinimidyl oleate (SSO) under HG+PA conditions. Lipid uptake assessed by BODIPY FL C_16_‐BSA F), cytotoxicity normalized to viability G), mitochondrial ROS production H), mitochondrial respiration I), mitochondrial membrane potential J), representative images of F‐actin by phalloidine staining K), and representative Oil red O staining and quantifications L)were assessed. Data are mean ± SEM. Scale bar: 4 and 20 µm in A (yellow bar represents 4 µm), 20 µm in C,H, and 10 µm in K and L. *n* = 6 mice per group and 30–34 glomeruli for A, *n* = 3 mice per group for B, *n* = 10 for C, *n* = 5 for D, E, and L, *n* = 3 for F, H–K, *n* = 4 for G. Statistical analysis was performed using one‐way or two‐way ANOVA test for A‐C, F, I and L, *t*‐test for D, E, G, H, J, and K. ^**^ *p* < 0.01, ^***^ *p* < 0.001, ns, not significant.

Based on our previous screening using the SGLT2i, which was reported to ameliorate podocyte lipotoxicity,^[^ ^19^ ^]^ we evaluated the impact of Dock5 deficiency on podocyte lipid metabolism. Substantially more severe lipid accumulation in podocytes was observed in cKO mice by co‐staining with adipophilin (a lipid droplet marker) and SYNPO under DKD conditions but not under basal conditions (**Figure** **4**A). Using liquid chromatography‐tandem mass spectrometry (LC‐MS/MS)‐based lipidomic analysis, we further revealed that the main species of lipid deposition were free fatty acids, triglyceride, ceramides and diacylglycerols (significantly increased), but not cholesterol esters and phosphatidylcholines (no statistical difference) (Figure 4B; Figure S3, Supporting Information), indicating that Dock5 deficiency primarily affected fatty acid metabolism in podocytes. Fatty acid metabolism mainly includes the processes of uptake, synthesis, and oxidation,^[^ ^23^, ^24^ ^]^ therefore these processes were measured. Knockdown of Dock5 in podocytes significantly elevated fatty acid uptake, which was proven by three different fatty acid labeling methods, i.e., Alexa‐labeled BSA, BODIPY FL C_16_ (fluorescent analog of PA) labeled BSA and ^13^C‐labeled PA (Figure 4C–E). Whereas, no significant alterations were observed in the fatty acid de novo synthesis and oxidation in si‐DOCK5 podocytes (Figure S4A,B, Supporting Information). These findings suggested that Dock5 mainly affects fatty acid import by podocytes. In podocytes, fatty acid uptake occurs mainly through two routes: the first is endocytosis by lipid‐binding G protein‐coupled receptors (GPCRs) or macropinocytosis, and the second is transported into cells by protein‐mediated mechanisms, which mostly rely on CD36.^[^ ^24^, ^25^, ^26^, ^27^ ^]^ Dock5 Knockdown did not alter the expression of lipid‐binding GPCRs, including free fatty acid receptor (FFAR)1, FFAR2, and FFAR3 (Figure S5A, Supporting Information). Additionally, repression of another major endocytic process, micropinocytosis, by wortmannin (a PI3K inhibitor to block macropinocytosis) did not block si‐Dock5 upregulated fatty acid import (Figure S5B, Supporting Information), which indicated that Dock5 regulation of fatty acid uptake was not mainly through endocytosis. While CD36 inhibitor Sulfo‐N‐succinimidyl oleate (SSO) obviously eliminated Dock5 loss‐induced fatty acid uptake (Figure 4F), suggesting that Dock5 deficiency enhanced fatty acid uptake primarily through the CD36‐regulated protein‐mediated route.

Then, the most important is whether CD36 regulates fatty acid uptake in relation to Dock5 deficiency‐induced podocyte damage. The CD36 inhibitor, SSO, significantly abolished Dock5 deficiency‐induced cytotoxicity, mitochondrial dysfunction, and actin cytoskeleton remodeling in podocytes (Figures 4G–K and 3G–K). Moreover, Dock5 silencing‐induced lipid accumulation was abrogated as well (Figure 4L). Taken together, these results suggested that Dock5 deficiency‐induced podocyte injury predominantly via promoting CD36‐mediated fatty acid uptake.

Additionally, as a guanine exchange factor for Rac family small GTPase 1 (Rac1), Dock5 has been documented to regulate Rac1.^[^ ^11^ ^]^ Thus, we assessed whether Rac1 also participates in Dock5‐regulated podocyte lipotoxicity. The results showed that after knocking down Rac1 (AAV2‐shRac1), Dock5 deficiency exacerbated KW/BW, UACR, glomerular pathology, podocyte loss, and lipid accumulation was not significantly altered (Figure S6A–G, Supporting Information; Figure 3A–F). These results indicated that the Dock5 deficiency exacerbated podocyte injury is not through Rac1.

### 2.5 Dock5 Modulates CD36 by Regulating LXRα in an m^6^A‐Dependent Way

**Figure 5:**
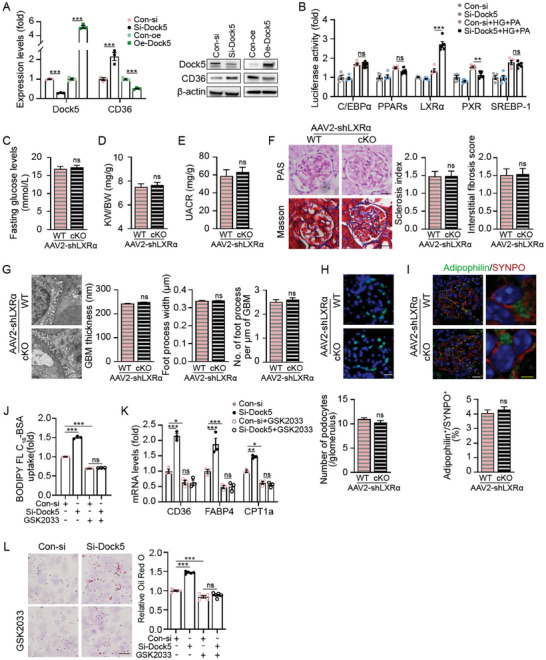
Dock5 regulates CD36‐mediated podocyte fatty acid uptake through LXRα. A) Expressions of indicated mRNA (left) or protein (right) in HG and PA‐treated podocytes transfected with Dock5‐specific siRNA or overexpression plasmids. B) Podocytes were transfected by Dock5‐specific siRNA with or without HG+PA. The luciferase reporter activities of indicated transcriptional factors were detected by the luciferase reporter assay. C–E) Dock5 cKO and its WT littermates were induced DKD by HFD feeding combined with STZ injection. LXRα was suppressed by AAV2‐Nphs1‐shLXRα injection (5 × 10^11^ vector genomes/mouse, tail vein). Fasting glucose levels C), KW/BW D), and UACR E) were detected. F) Representative images of PAS and Masson staining in specific mouse groups were shown, and their quantifications were listed. G) Representative TEM images and quantifications of GBM thickness, foot process width, and the number of foot processes in mice from indicated groups. H) Representative images and quantifications of WT1 staining in specific mouse groups were shown. I) Representative IF images and quantifications showing lipid accumulation in podocytes (co‐staining adipophilin (green) with SYNPO (red)). J–L) Podocytes were treated by HG+PA together with or without LXRα antagonist GSK2033. Lipid uptake J), mRNA expressions of the indicated gene K), and lipid accumulations L) were measured by BODIPY FL C_16_‐BSA, qRT‐PCR, and Oil red O staining, respectively. Data are mean ± SEM. Scale bar: 20 µm in F and H, 1 µm in G, and 4 and 20 µm in I (yellow bar represents 4 µm), 10 µm in L. *n* = 3 for A, J, and K, *n* = 5 for B and L, *n* = 4 mice per group for C–I, *n* = 41–59 glomeruli for F, *n* = 10 images per mouse for G. Statistical analysis was performed using one‐way ANOVA test for A, B, J–L, *t*‐test for C–I. ^*^ *p* < 0.05, ^**^ *p* < 0.01, ^***^ *p* < 0.001, ns, not significant.

Next, we looked into the mechanism underlying the Dock5‐regulated CD36. Both the mRNA and protein expression of CD36 were regulated by Dock5 manipulation, indicating that Dock5 modulated CD36 might occur at the transcriptional level (**Figure** **5**A). Several transcription factor response elements binding sites have been identified in the promoter region of the CD36 gene, such as binding sites for CCAAT enhancer binding protein α (C/EBP)α, PPARs, LXRα, pregnane X receptor (PXR), and sterol regulatory element binding transcription factor 1 (SREBP‐1). Therefore, transcription factors were reported as the most important regulators of CD36 transcription.^[^ ^28^, ^29^ ^]^ We then performed the transient transfection assay with the above transcription factors, and the results showed Dock5 knockdown increased luciferase reporter gene activity in the presence of LXRα, but not C/EBPα, PPARs, PXR, and SREBP‐1 (Figure 5B). Then, to assess if LXRα was responsible for the effects of Dock5 in podocytes, podocyte‐specific interference of LXRα in Dock5 cKO mice was conducted by AAV2 ‐Nphs1 (podocyte‐specific promoter)‐shLXRα under the DKD condition. Knocking down LXRα in podocytes significantly attenuated Dock5 deficiency‐induced a series of alterations, such as KW/BW, UACR, glomerular pathology, podocyte foot process broadening and effacement, podocyte loss and lipid accumulation (Figures 3, 5, and 4A). Moreover, the LXRα antagonist GSK2033 abolished Dock5‐knockdown induced fatty acid uptake, CD36 and its downstream gene expressions, and lipid overload in cultured podocyte cell lines (Figure 5J–L). As for how LXRα affected CD36 expression, a previous study identified a DR‐7 type nuclear receptor response element in the promoter region of the CD36 gene, which is responsible for mediating the activation of CD36 transcription by LXRα in hepatocytes.^[^ ^30^ ^]^ In agreement, our chromatin immunoprecipitation (ChIP) assays showed that LXRα agonist T0901317 could enhance the occupancy of LXRα on the DR‐7 element encompassing CD36 promoter region in podocytes (Figure S7A, Supporting Information). Further luciferase activity analysis revealed that CD36 (DR‐7) reporter was activated by T0901317 in podocytes, and this activation was abrogated by introducing mutations to the DR‐7 region (Figure S7B, Supporting Information), suggesting that LXRα activated CD36 gene transcription through directly binding to its promoter region in podocytes. These in vivo and in vitro results suggested that Dock5 regulated CD36 mainly via transcription factor LXRα in podocytes.

**Figure 6:**
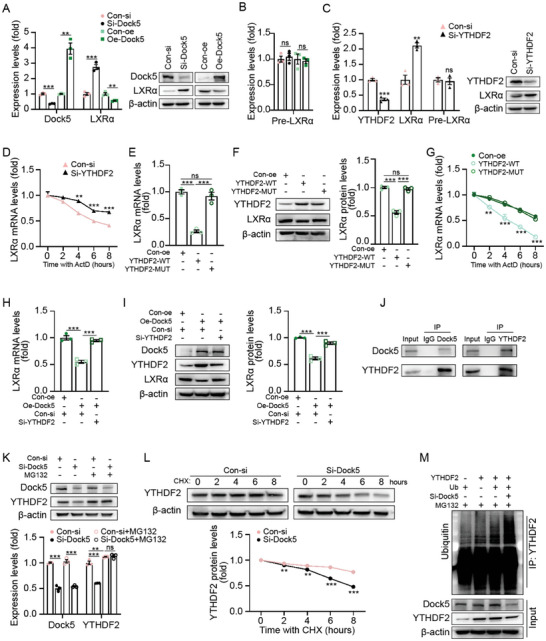
Dock5 modulates the degradation of LXRα mRNA through m^6^A‐dependent machinery. A,B) Expressions of indicated mRNA (left panel of A, and B) or protein (right panel of A), in HG and PA‐treated podocytes transfected with Dock5‐specific siRNA or overexpression plasmids. C) Expressions of indicated mRNA (left) or protein (right) in HG and PA‐treated podocytes transfected with YTHDF2‐specific siRNA. D) Half‐life of LXRα mRNA in HG and PA‐treated podocytes upon silencing YTHDF2 with the treatment of Actinomycin D (ActD) (5 µm) before total mRNAs were collected at different time points. E–G) HG and PA‐treated podocytes were transfected by overexpressing plasmids of YTHDF2 wild‐type (WT) or m^6^A recognition defective YTHDF2 mutant (W432A/W486A/W491A, MUT) plasmids, mRNA expression (E), protein expression F), and mRNA half‐life G) of LXRα were assessed. For the half‐life assay, ActD was added to inhibit the transcription. H,I) HG and PA‐treated podocytes were transfected by Dock5 overexpressing plasmids with YTHDF2‐specific siRNA or control siRNA. mRNA expression H) and protein expression I) of LXRα were assessed. J) Dock5 interacts with YTHDF2 by the reciprocal co‐IP assays in HG and PA‐treated podocytes. K) HG and PA‐treated podocytes were transfected with Dock5‐siRNA or control siRNA combined with or without the proteasome inhibitor MG132. L) HG and PA‐treated podocytes were transfected with Dock5‐siRNA or control siRNA with cycloheximide (CHX) and harvested at different time points as indicated, protein expression and quantifications of YTHDF2 were assessed by immunoblotting. M) HG and PA‐treated podocytes were transfected with indicated plasmids and siRNA, and the ubiquitination of YTHDF2 was assessed by immunoblotting. Data are mean ± SEM. *n* = 3 for A–I, K, and L. Statistical analysis were performed using a one‐way or two‐way ANOVA test for D–I, K, and L, *t*‐test for A–C. ^**^ *p* < 0.01, ^***^ *p* < 0.001, ns, not significant.

Next, we investigated the mechanism by which Dock5 regulates LXRα. Dock5 manipulation altered the abundance of LXRα at both mRNA and protein levels (**Figure** **6**A), which indicates that Dock5 may modulate LXRα at the transcriptional level. However, a recent study^[^ ^31^ ^]^ reported that LXRα can be regulated in a post‐transcriptional way, i.e., m^6^A‐mediated mRNA decay, which also leads to alterations of both mRNA and protein expressions. Therefore, to observe that Dock5 regulated LXRα at the transcriptional or post‐transcriptional level, the abundance of LXRα pre‐mRNA was assessed. LXRα pre‐mRNA was barely changed by Dock5 manipulation (Figure 6B), indicating that Dock5 may function post‐transcriptionally to regulate LXRα mature mRNAs. As per the study we mentioned above,^[^ ^31^ ^]^ LXRα mature mRNA could undergo m^6^A‐mediated mRNA decay via the interaction with m^6^A “reader” YTH N6‐methyladenosine RNA binding protein F2 (YTHDF2). We then examined if Dock5 regulates LXRα in podocytes through the YTHDF2‐mediated m^6^A pathway. RNA immunoprecipitation analysis showed that the YTHDF2 protein could bind to LXRα mRNAs (Figure S8A, Supporting Information). YTHDF2 knockdown increased LXRα protein and mRNA levels without affecting its pre‐mRNA (Figure 6C), and inhibited the decay rate of LXRα mRNAs, which was detected when treating podocytes with transcription inhibitor actinomycin D (Figure 6D). Moreover, we adopted the YTHDF2 plasmid with mutations of the m^6^A recognition sites (W432A, W486A, and W491A)^[^ ^32^ ^]^ to examine whether YTHDF2 regulated LXRα expression through m^6^A methylation. As expected, m^6^A‐recognition of defective YTHDF2 (YTHDF2‐MUT) failed to decrease the levels of mRNAs and proteins of LXRα as compared with YTHDF2‐WT (Figure 6E,F). Meanwhile, the accelerated mRNA decay of LXRα under the YTHDF2‐WT transfection condition was not observed when overexpressing YTHDF2‐MUT alone (Figure 6G). To assess the intermediate role of YTHDF2 in Dock5‐regulated LXRα expression, we knocked down YTHDF2 in Dock5‐overexpressed podocytes and found that the ablation of YTHDF2 abrogated Dock5 suppressed LXRα expression (Figure 6H,I). Furthermore, we employed CRISPRCas9‐based m^6^A editing systems to determine whether the addition of the methyl groups to LXRα mRNA per se is responsible for Dock5/YTHDF2 regulated LXRα mRNA destabilization. To guide RNA demethylation at the designated transcriptomic locus, the endonuclease dead Cas9 protein (dCas9) was fused to the RNA demethylase alkB homolog 5 RNA demethylase (ALKBH5). dCas9 fused with ALKBH5‐H204A (histidine 204 was mutated to alanine to inactivate the enzyme) was used as the inactive control. In addition, we generated two pairs of single‐guide RNAs (sgRNAs) and corresponding protospacer adjacent motifs (PAMers) to target adenosine at the putative m^6^A sites within the 3′UTR region of LXRα mRNA. Within each pair, one sgRNA was located 3 bp to the target adenosine (sgLXRα‐A or sgLXRα‐B), and the other one, which served as an ineffective control, was designed 10 bp away from the former (sgLXRα‐A‐Ctrl or sgLXRα‐B‐Ctrl) (Figure S8B, Supporting Information). Increased abundance along with an inhibited decay rate of LXRα mRNAs was only observed when both the functional sgRNAs and ALKBH5 were successfully transfected into podocytes, in contrast to the transfection with ineffective control sgRNAs and enzymatically inactive ALKBH5‐H204A (Figure S8C,D, Supporting Information). Taken together, these results indicated that Dock5 restrained LXRα expression through YTHDF2‐mediated m^6^A‐dependent mRNA decay in podocytes, and m^6^A modification on LXRα mediated its mRNA degradation.

To decipher the molecular mechanism underlying the Dock5‐mediated regulation of YTHDF2, we performed reciprocal co‐immunoprecipitation assays and verified that Dock5 binds to YTHDF2 in podocytes (Figure 6J). Increased YTHDF2 protein levels were observed after Dock5 overexpression in Figure 6I (lane one compared with lane two); interestingly, YTHDF2 mRNA levels were not affected (Figure S9, Supporting Information). We previously revealed the role of Dock5 in regulating protein stability in keratinocytes,^[^ ^17^ ^]^ and others have shown that the YTHDF2 protein could be degraded by the proteasomal pathway^[^ ^32^, ^33^ ^]^; we then sought to determine whether Dock5 could modulate YTHDF2 protein stability in podocytes. By introducing Dock5 siRNAs into podocytes, we found that the protein level of YTHDF2 dramatically declined upon Dock5 ablation, and this reduction was completely restored by treatment of cells with the proteasome inhibitor MG132 (Figure 6K). Additionally, the knockdown of Dock5 shortened the half‐life of the YTHDF2 protein (Figure 6L). In agreement with these results, we also showed that si‐Dock5 drastically enhanced YTHDF2 ubiquitination (Figure 6M). Collectively, these findings demonstrated that Dock5 deficiency prompted ubiquitination‐mediated proteolytic degradation of YTHDF2 therefore affecting the stability of m^6^A modified LXRα mRNA in podocytes.

### 2.6 Restoration of Dock5 Expression Alleviated Podocyte Injury in Proteinuric Kidney Diseases

**Figure 7:**
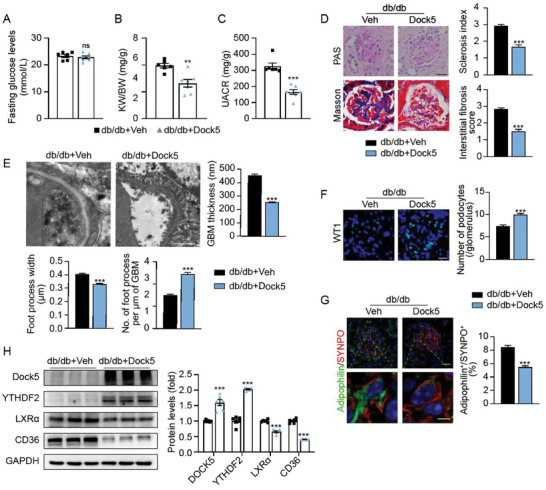
Restoring Dock5 expression alleviates podocyte lipid accumulation, injury, and glomerular pathologies in db/db diabetic mice. A–G) db/db mice were injected in situ with adenovirus expressing Dock5 (Ad‐Dock5) or vehicle (Ad‐Veh), and fasting glucose levels A), KW/BW B), and UACR C) were assessed. D) Representative images of PAS and Masson staining in specific mouse groups were shown, and their quantifications were listed. E) Representative TEM images and quantifications of GBM thickness, foot process width, and the number of foot processes in mice from indicated groups. F) Representative images of WT1 staining in specific mouse groups were shown, and their quantifications were listed. G) Representative IF images showing lipid accumulation in podocytes (co‐staining adipophilin (green) with SYNPO (red)), and their quantifications are shown below. H) Expression of indicted protein and quantifications were measured in mice from specified groups. Data are mean ± SEM. Scale bar: 20 µm in D,F, 1 µm in E, 4 and 20 µm in G (yellow bar represents 4 µm). *n* = 6–7 mice per group for A–H, *n* = 61–104 glomeruli for D and F, *n* = 10 images per mouse for E, *n* = 29–34 glomeruli for G. Statistical analysis was performed using *t*‐test for A–H. ^**^ *p* < 0.01, ^***^ *p* < 0.001, ns, not significant.

Finally, we investigated the effects of rescuing Dock5 expression on proteinuric kidney diseases. Dock5‐overexpressed mice were successfully generated by in situ injection of adenoviral vectors expressing mouse Dock5 (Ad‐Dock5) at four independent points in each kidney (Figure S10, Supporting Information), according to previous studies.^[^ ^34^, ^35^ ^]^ Although no changes were observed in the glucose levels of Dock5‐overexpressed DKD mice, the KW/BW and UACR were significantly reduced (**Figure** **7**A–C). Moreover, glomerular pathology, podocyte foot process broadening and effacement, podocyte loss, and lipid accumulation were markedly improved by Ad‐Dock5 treatment (Figure 7D–G). In addition, corroborating our previous mechanistic findings, the increased expression of YTHDF2, and decreased LXRα and CD36 were observed with Dock5 replenishment (Figure 7H). These findings indicate that rescue of Dock5 deficiency may mitigate the progression of proteinuric kidney diseases, and is a promising therapeutic approach for this type of disease.

## 3 Discussion

Proteinuric kidney diseases are the leading cause of ESRD worldwide, and despite several therapies that have been applied clinically for decades, there is still a large residual risk for the onset and progression of these diseases. Recently, emerging evidence has suggested that disorders of lipid metabolism and lipid accumulation in podocytes are pivotal to the pathogenesis of renal damage in proteinuric kidney diseases.^[^ ^24^, ^36^, ^37^ ^]^ For example, Fu et al., revealed that increased junctional adhesion molecule‐like protein (JAML) induces podocyte lipid accumulation and promotes renal injury in proteinuric kidney diseases.^[^ ^36^ ^]^ Although evidence is continuously being gathered that shows a critical correlation between disruptions in podocyte lipid metabolism and proteinuric kidney diseases, the underlying mechanism remains largely unclear. In this study, we demonstrated a heretofore uncovered role of podocyte predominantly expressed protein Dock5, which may have an important function in podocyte lipid accumulation. The expression of Dock5 was reduced in both proteinuric kidney disease patients and mouse models. Podocyte‐specific deletion of Dock5 exacerbates podocyte injury and glomerular pathology in proteinuric kidney disease. Interestingly, the effects of Dock5 deficiency were predominantly observed under disease conditions, but not under basal conditions, indicating that genetic determinants need to cooperate with environmental factors to drive proteinuric kidney disease initiation and progression. This phenomenon has also been observed in several pathogenic genes related to kidney disease, such as JAML^[^ ^36^ ^]^ and apolipoprotein L1.^[^ ^38^ ^]^

As an exchange factor for the RhoGTPases, Dock5 was originally identified as a regulator of osteoclastogenesis.^[^ ^11^, ^16^, ^39^ ^]^ Our previous study showed that Dock5 contributes to the regulation of wound healing^[^ ^17^ ^]^ and hepatic insulin resistance.^[^ ^18^ ^]^ Here, we uncovered a critical role in podocyte lipid metabolism, indicating the multifunctionality of Dock5. Notably, we found that Dock5 regulates podocyte lipid metabolism through CD36. As a ubiquitously expressed protein, CD36 has been observed to manage lipid metabolism in several cell types, including hepatocytes and adipocytes.^[^ ^40^ ^]^ Therefore, the potential effects of Dock5 on lipid metabolism in these tissues should be explored in future studies.

Our results suggested that Dock5 regulates CD36 through transcription factor LXRα in podocytes. CD36 was reported to be regulated by transcription factors C/EBPα and PPARs in kidneys by previous studies,^[^ ^28^ ^]^ and here we demonstrated that it could also be regulated by LXRα. Interestingly, the modulation of LXRα on CD36 has only been previously reported in hepatocytes and contributed to the hepatic lipid metabolism disorder and steatosis.^[^ ^30^ ^]^ Therefore, this further implies our previous inference that Dock5 may also have a potential role in hepatic lipid metabolism.

In addition, we revealed that Dock5 modulated LXRα in an m^6^A‐dependent way. m^6^A is the most common post‐transcriptional RNA modification, and its functions in regulating the cell cycle, cell differentiation and reprogramming, circadian rhythm maintenance, and stress responses have been unveiled in recent years.^[^ ^41^, ^42^ ^]^ Our results suggest that m^6^A may also play an important role in fatty acid metabolism in podocytes. Moreover, the modulation of LXRα by m^6^A was previously reported in brain lipid metabolism,^[^ ^31^ ^]^ which further highlights the potential role of Dock5 in lipid metabolism beyond the kidneys.

Our findings suggest that Dock5 may serve as a potential therapeutic target for proteinuric kidney diseases. Although several therapeutic strategies, such as antihypertension, RAAS inhibition, low‐protein diets, and nutritional supplementation, have been applied for the treatment of proteinuric kidney diseases for decades, the prevalence of ESRD is continuously increasing worldwide. Our results suggest that the restoration of Dock5 deficiency significantly attenuates podocyte injury and glomerular pathology, indicating that Dock5 may be a potential target for the treatment of proteinuric kidney diseases. Additionally, the size of the Dock5 gene is >5Kb, which may have affected the selection of vectors for future gene therapy. In the current study, we chose to overexpress Dock5 using adenovirus instead of AAV because the vector genome length of AAV is below 4.7–5Kb.^[^ ^43^, ^44^ ^]^

## 4 Conclusion

In summary, our study demonstrated a novel role of Dock5 in podocyte lipotoxicity and proteinuric kidney disease, which is mainly through modulating fatty acid uptake by the LXRα/CD36 signaling pathway, and it may also serve as a promising therapeutic target.

## 5 Experimental Section

### Human Samples

Human renal tissues were obtained from 6 DKD and 7 nondiabetic patients who underwent partial or radical nephrectomy for T1 renal cancer (the tumor was found only in the kidney and was 7 cm or smaller). Both biochemical examinations (including UACR > 30 mg g^−1^) and histological analyses (according to the Renal Pathology Society's Pathological Classification of Diabetic Nephropathy) were conducted to confirm the diagnosis of DKD. All samples were collected according to a protocol approved by the Ethics Committee of the Second Affiliated Hospital of Army Medical University (Institutional Review Board–approved protocol number 2016‐056‐01). All the procedures were conducted in compliance with the principles of the Declaration of Helsinki. Informed consent was obtained from all the participants. Clinical information was retrieved from the medical records and is summarized in Table S3 (Supporting Information).

### Mice and Treatment

Male C57BL/6J, BALB/c, homozygous db/db (C57BLKS/J‐LepR), and heterozygous db m^−1^ mice were purchased from GemPharmatech Co., Ltd, Jiangsu, China. Podocyte‐specific Dock5 knockout mice were generated by crossbreeding a previously described Dock5 conditional allele (Dock5^fl/fl^, C57BL/6JGpt background) with podocin (nphs2)‐Cre mice (C57BL/6J background). Both Dock5^fl/fl^ and podocin‐Cre were purchased from GemPharmatech Co., Ltd, Jiangsu, China. Genotyping was performed using genomic DNA isolated from the tails of mice aged 2–3 weeks. Flox genotyping primers (forward 5′‐AAGTGTTCAGTGTGGATTGACTCAGAG‐3′; reverse 5′‐AACACCACACATAAGGTGACCTTGAG‐3′’), and Cre genotyping primers (forward 5′‐CGGTTATTC AACTTGCACCA‐3′; reverse 5′‐GCGCTGCTGCTCCAG‐3′) was used. Only male mice were used for experiments, except in the case of primary cell isolation.

Considering that mice with a C57BL/6J background may be resistant to STZ‐induced DKD, DKD in podocyte‐specific Dock5 knockout mice by HFD/STZ treatment was induced.^[^ ^36^, ^45^ ^]^ Six‐week‐old male Dock5^fl/fl^‐Cre^+^ mice and their littermate Dock5^fl/fl^‐Cre^−^ mice were fed a high‐fat diet (HFD, 60 kcal% from fat, Research Diets, D12492) or normal chow diet (NCD) for 6 weeks and then injected with low‐dose STZ (intraperitoneal, 50 mg k^−1^g) for 3 consecutive days. After STZ injection mice were continuously fed an HFD or NCD for 16 weeks. Mice with fasting blood glucose (FBG) levels ≥250 mg dl^−1^ were considered diabetic and used for further study. ADR‐induced FSGS mice were generated as previously described.^[^ ^46^ ^]^ For knocking down LXRα in vivo, AAV2‐Nphs1‐shLXRα were generated by OBiO Technology (Shanghai, China) and were injected into mice via the tail vein at a dose of 5 × 10^11^ vector genomes per mouse. To rescue Dock5 expression in the kidney of DKD mice, adenovirus vectors expressing Dock5 (Ad‐Dock5) and a control vector (Ad‐Veh) were generated using GENECHEM (Shanghai, China). db/db and STZ DKD mice received in situ renal injection with Ad‐Dock5 or Ad‐Veh at 4 independent locations (20 µL each, 1 × 10^10^ PFU 80 uL^−1^) bilaterally as previously described.^[^ ^34^ ^]^ A total of 2 × 10^10^ PFU of virus was injected per mouse, and 4 weeks later, the mice were euthanized.

All animal study protocols were approved by the Laboratory Animal Welfare and Ethics Committee of the Army Medical University (AMUWEC20224451), and all procedures followed the National Institutes of Health Guide for the Care and Use of Laboratory Animals. All mice were housed under a standard 12 h dark/light cycle at a constant temperature of 22 ± 1 °C with ad libitum access to water and food. Age‐matched littermates served as controls which were randomly divided into groups. Investigators were blinded to the allocation of groups when conducting renal function, histology, and pathology analyses. The exclusion criteria were based on animal well‐being before the start of the experiments. The number of mice used in the study was determined according to previous reports and is indicated for each experiment in the figure legends.

### RNA‐Sequencing Analysis

Total RNA from podocytes treated with SGLT2i and podocytes isolated from DKD mice was extracted using an MJZol total RNA extraction kit (Majorbio, China). RNA integrity and genomic DNA contamination were detected using denaturing agarose gel electrophoresis and an Agilent 2100 Nano (Agilent Technologies). RNA sequencing was performed by Majorbio Bio‐Pharm Technology Co. Ltd. (Shanghai, China) using an Illumina NovaSeq 6000 platform. Data were analyzed using the I‐Sanger online platform.

### Cell Culture and Treatment

The conditionally immortalized mouse podocyte cell line (MCP5) was purchased from the BeNa Culture Collection (BNCC342021, Henan, China). Cell line identification and mycoplasma contamination were performed by the same company. Podocytes were cultured in RPMI 1640 supplemented with 10% fetal bovine serum (FBS, ExCell Bio) and 100 U mL^−1^ penicillin plus 0.1 mg mL^−1^ streptomycin at 33 °C for proliferation, and were then thermos‐shifted to 37 °C for differentiation.

Primary mouse podocytes were isolated as described previously.^[^ ^46^ ^]^ In brief, Dynabeads M‐450 Tosylactivated (Invitrogen, #14013) was perfused from the mouse aorta with Hank's balanced salt solution (HBSS), and the kidneys were harvested and minced in a tissue digestion solution containing 1 mg mL^−1^ collagenase I (Sigma‐Aldrich, C0130) and 100 U mL^−1^ DNAse I (Sigma‐Aldrich, D5025). Tissue lysis was then passed through a 100 µm cell strainer (Falcon, 352360) and glomeruli were collected by using a magnet. These glomeruli were then cultured on precoated collagen I dish for 5 days, trypsinized, and filtered through a 40 µm cell strainer (Falcon, 352340) to prepare the primary podocytes. Primary mouse kidney tubular epithelial, glomerular endothelial, and mesangial cells were purchased from ProCell Life Science & Technology Co. Ltd. (Hubei, China).

For gene knockdown and overexpression experiments, cells were plated and transfected with siRNA oligonucleotides using Lipofectamine RNAiMAX (Thermo Fisher, #13778150) or plasmids using Lipofectamine 3000 (Invitrogen, #L3000015). Dock5‐, LXRα‐, YTHDF2‐specific, and control siRNAs were purchased from Qiagen (Shanghai, China) and RiboBio (Guangzhou, China). Dock5, YTHDF2 (WT and mutant), and control vector plasmids were generated by Sino Biological, Inc. (Beijing, China). Luciferase activity was assessed using a luciferase assay system according to the manufacturer's instructions (Promega). Luciferase reporter plasmid of PPARs, LXRα, PXR, SREBP‐1, CD36WT, CD36MUT, and control Renilla vectors were generated from Yeasen Biotechnology (Shanghai), Bioegene, and Promega.

For inhibiting YTHDF2 protein degradation, cells were incubated with the proteasome inhibitor MG132 (dissolved in DMSO at a final concentration of 25 µm) for 6 h. The half‐life of the YTHDF2 protein was determined according to the previous protocol.^[^ ^47^ ^]^ In brief, cells were treated with cycloheximide (CHX, 25 µm) for blocking protein synthesis, and then cell lysates were collected at different time points and subjected to immunoblot analysis.

### Immunofluorescence, MitoSOX, and JC‐1 Staining

Immunofluorescent staining of mouse and human kidney tissue was performed using Tissue‐Tek O.C.T. (Sakura, Japan) embedded cryosections or paraffin‐embedded sections, respectively. 6–8 µm for cryosections and 4 µm for paraffin sections were incubated with different primary antibodies against the following proteins: Dock5 (Bioegene, Shanghai, China), synaptopodin (Novus, NBP2‐39100), Wilms Tumor protein (WT1, Abcam, ab89901), adipophilin (Proteintech, 15294‐1‐AP) and phalloidin (Invitrogen, A34055), and subsequently incubated with secondary antibody (Invitrogen). Isotype‐matched IgG was used as a negative control in preliminary studies. Reactive oxygen species were detected by staining the podocytes with the MitoSOX Red superoxide indicator (Invitrogen, M36008) according to the manufacturer's instructions. Mitochondrial membrane potential in podocytes was assessed by JC‐1 Dye according to the manufacturer's instructions.

### Western Blot Analysis

Western blot analysis was performed as previously described.^[^ ^47^ ^]^ Briefly, tissues and cells were lysed in RIPA buffer containing a protease inhibitor cocktail (Beyotime Biotechnology, Shanghai, China). Protein concentrations were assessed using a BCA protein assay. Proteins were separated by SDS‐PAGE, transferred onto PVDF membranes, and immunoblotted for the following antibodies: Dock5 from Bioegene, LXRα, and YTFDH2 from Proteintech.

### UACR and Renal Histopathology

The UACR was determined according to the previous report.^[^ ^46^ ^]^ Urea albumin and creatinine levels were measured using commercial assay kits (Jiancheng Biochemical Co., Jiangsu, China). PAS, Masson's trichrome, and Oil Red O staining were performed on paraffin‐embedded sections or OCT‐embedded cryosections, as in previous reports.^[^ ^46^, ^47^ ^]^ PAS‐stained kidney sections were scored to assess the extent of mesangial expansion using a semiquantitative method, as previously described.^[^ ^48^ ^]^ An arbitrary scale from 0 to 4 was determined based on the percentage of glomerular lesions as follows: 0, no lesions; 1–4, <25%, 25–50%, 50–75%, and >75% of the glomerulus. Masson‐stained sections were scored to evaluate interstitial fibrosis^[^ ^49^ ^]^ using a similar arbitrary scale from 0 to 4. Oil‐Red O‐stained sections were analyzed using ImageJ software (National Institutes of Health, NIH, Bethesda, MD) to quantify lipid accumulation.

### Ultra‐Performance Liquid Chromatography (UPLC) and Mass Spectrometry (MS/MS) Analysis

Renal cortical tissues were prepared for lipidomic analysis as previously described.^[^ ^36^ ^]^ Samples were snap‐frozen in liquid nitrogen and stored at −80 °C. UPLC‐MS/MS analysis was performed by Metware Biotechnology Co., Ltd (Wuhan, China).

### Cytotoxicity and Viability Analysis

The cytotoxicity and viability of the podocytes were assessed using the ApoTox‐Glo Triplex Assay (Promega, G6321) according to the manufacturer's instructions. The luminescence was measured using a SpectraMax iD3 multi‐mode microplate reader (Molecular Devices).

### Fluorescently Labeled Fatty Acid Internalization Assay

Fatty acid internalization in podocytes was assessed using the Alexa‐labeled BSA and BODIPY FL C16 assays according to a previous report.^[^ ^50^ ^]^ To assess the uptake of different lipids, podocytes were treated by Alexa Fluor 488‐labeled BSA (100 µg mL^−1^, Bioss, Beijing, China), and then photographed by Confocal Microscope ZEISS LSM900. BODIPY FL C16 (D3821, Invitrogen) labeling experiments were performed according to the manufacturer's instructions. Cell samples were suspended and injected into a flow cytometer, and data were analyzed using FlowJo V10.7 software (BD Biosciences, MA, USA).

### Stable Isotope–Labeled Lipid Uptake and Lipogenesis Analysis

The ^13^C labeling PA uptake and the ^13^C labeling glucose‐derived de novo lipogenesis were determined according to the previously described.^[^ ^51^, ^52^ ^]^ To assess lipid uptake, the isolated podocytes were incubated in a fresh medium for 6 h, and then treated with a known concentration of [U‐13C]palmitic acid for 3 h before the medium was sampled. Calibration standard solutions were prepared by stepwise dilution of the stock solution, which was diluted with the standard substances (final concentration of 1 mmol L^−1^). For de novo lipogenesis analysis, podocytes were treated with 25 mm [U‐13C]glucose for 12 h and then were washed twice with PBS, and gently scraped from T flasks with 1 mL of precooled methanol: water (80%: 20%, v/v) mixture. The samples were analyzed using LC MS analysis at Metware Biotechnology.

### Transmission Electron Microscopy (TEM)

Kidney samples were harvested and cut into small pieces of 1 mm^3^ in a fixative for TEM (Servicebio, Hubei, China), and then stored at 4 °C for transportation. The tissue samples were then submitted to Service Technology Co. LTD for washing, dehydration, resin penetration, and embedment according to standard procedures. Ultrathin sections were observed using a HITACHI HT7800 Transmission Electron Microscope. Ten images were obtained from each mouse for analysis, and the GBM thickness, foot process width, and number were analyzed using the ImageJ software.

### Seahorse‐Based Fatty Acid Oxidation (FAO) and Oxygen Consumption Rate (OCR) Analysis

FAO and OCR were assessed using an Agilent Seahorse XF extracellular flux analyzer (Agilent Technologies) according to a previously described method.^[^ ^46^, ^47^ ^]^ For FAO analysis, podocytes were plated on Seahorse assay plates and treated with Seahorse XF assay medium containing DMEM with 5.5 mm glucose and 0.5 mm carnitine according to the manufacturer's instructions from Seahorse Bioscience. A specific CPT‐1 inhibitor Etomoxir (ETO, 250 µm) was injected to evaluate the rate of FAO, and then a blocker of glycolysis and pyruvate oxidation 2‐deoxyglucose (2‐DG, 50 mm) was injected to assess the glucose utilization. For the OCR analysis, podocytes were seeded in Seahorse assay plates and treated with Seahorse XF assay medium. After obtaining the baseline OCR, the measurements were obtained after sequential injection of 5 µm oligomycin, 1.5 µm FCCP, and 5 µm rotenone.

### Chromatin Immunoprecipitation (ChIP)–qPCR Analysis

ChIP analysis was performed by using the commercial kit (Abcam, ab500) according to its protocols. Podocytes were collected and fixed in a final formaldehyde concentration of 1.1% at room temperature. The chromatin was sonicated into optimal fragments with lysis buffer and then incubated overnight with control IgG or anti‐HA antibodies (Cell Signaling Technology) at 4 °C. Protein A sepharose beads were used for antibody pulldown. Then the ChIP'd DNA was washed, reverse cross‐linked, and purified following the kit protocols. PCR primer sequences for ChIP‐qPCR are listed in Table S4 (Supporting Information).

### Quantitative Real‐Time PCR (qRT‐PCR)

qRT‐PCR analyses were performed as previously described, using the CFX Connect Real‐Time PCR Detection System with TB Green Premix Ex Taq II (RR820A, Takara). RNA was isolated from the kidneys and cells using TRIzol (Takara, 9109) and reverse‐transcribed into cDNA using the PrimeScript RT Reagent Kit (Takara, RR047A). The primer sequences are listed in Table S5 (Supporting Information).

### dCas9‐Based m^6^A‐Editing System Generation

Podocytes were co‐transfected with plasmids expressing dCas9 fusion protein and LXRα‐targeting or control sgRNAs at a mass ratio of 3:1 using Lipofectamine 3000. The corresponding PAMers were transiently delivered into podocytes using Lipofectamine 3000 on the following day. Seventy‐two hours after transfection, the podocytes were subjected to qRT‐PCR and mRNA stability assays. Sequences of control sgRNA, LXRα‐targeting sgRNAs, and PAMers were listed in Table S6 (Supporting Information).

### Statistical Analysis

Data are expressed as the mean ± standard error of the mean (SEM) or standard deviation (SD), as stated in the figure legends. The unpaired Student's *t*‐test, with no assumption of equal variance, was used for comparisons between the two groups. One‐way analysis of variation (ANOVA) with post hoc Tukey's test was used to determine the differences between multiple groups with one variable. Two‐way ANOVA, followed by Tukey's post hoc test, was performed to compare multiple groups with more than one variable. The sample size (n) for each experiment was stated in the figure legends. Analyses were performed using the GraphPad Prism software (version 8.0, GraphPad Software, CA, USA). Statistically, significance was defined as a two‐sided *p*‐value <0.05.

## Conflict of Interest

The authors declare no conflict of interest.

## Author Contributions

H.Q., X.L., J.Z., X.X., and L.L. contributed equally to this work. H.Q., Y.Z., XF.L., QS.H., and L.L. designed and conducted in vivo and in vitro experiments, acquisition of data, and performed statistical analysis; H.Q., X.L., L.Z., Y.W., X.X., G.Y., and X.G. analysis and interpretation of data; J.Z. and Q.H. performed animal studies and helped with data analysis; Y.Z., and H.Q. drafting of the manuscript; G.L., Q.Y., H.Z., and Y.Z. critical revision of the manuscript for important intellectual content; H.Z. and Y.Z. were the guarantors of this work and, as such, had full access to all the data in the study and took responsibility for the integrity of the data and the accuracy of the data analysis.

## Supporting information

Supporting Information

Supplemental Table 1

Supplemental Table 2

Supplemental Table 3

## Data Availability

The data that support the findings of this study are available on request from the corresponding author. The data are not publicly available due to privacy or ethical restrictions.
