# Supplementary material for: Dock5 Deficiency Promotes Proteinuric Kidney Diseases via Modulating Podocyte Lipid Metabolism
Source: Adv Sci (Weinh). 2023 Dec 31;11(11):2306365. doi: 10.1002/advs.202306365 (PMC10953540; doi:10.1002/advs.202306365)
Supplement: Supplementary file 1 — Supporting Information [file ADVS-11-2306365-s004.pdf]

ADVANCED SCIENCE
Open Access

Supporting Information

for *Adv. Sci.*, DOI 10.1002/advs.202306365

Dock5 Deficiency Promotes Proteinuric Kidney Diseases via Modulating Podocyte Lipid Metabolism

Hua Qu, Xiufei Liu, Jiaran Zhu, Xin Xiong, Lu Li, Qingshan He, Yuren Wang, Guojun Yang, Linlin Zhang, Qingwu Yang, Gang Luo, Yi Zheng* and Hongting Zheng**

Supporting Information

Supporting contents

Figure S1. Dock5 shows a podocyte predominant expression pattern in line with published single-cell RNA sequencing datasets.

Figure S2. Dock5 expression is specifically deleted from podocytes.

Figure S3. Dock5 deletion promotes toxic lipid accumulation, but does not influence cholesterol ester and phosphatidylcholines accumulation.

Figure S4. Dock5 has no effects on fatty acid de novo synthesis and oxidation.

Figure S5. Dock5 deficiency induced fatty acid uptake is endocytosis-independent.

Figure S6. Dock5 deficiency exacerbates podocyte injury and lipotoxicity may independent of Rac1.

Figure S7. LXRα activates CD36 gene transcription via binding to its promoter region.

Figure S8. The m^6A modification per se expedites the degradation of LXRα mRNA.

Figure S9. Dock5 has no impact on YTHDF2 mRNA expression.

Figure S10. Dock5 expression is rescued in podocyte from db/db diabetic mice by Ad-Dock5 treatment.

Table S1. Differentially expressed gene dataset of podocytes treated by SGLT2i.

Table S2. Differentially expressed gene dataset of podocytes isolated from DKD mice.

Table S3. Clinical and laboratory characteristics of all participants.

Table S4. List of primers for ChIP-qPCR.

Table S5. List of primers.

Table S6. sgRNA oligos and PAMers used in CRISPR-based m^6A-editing tools.

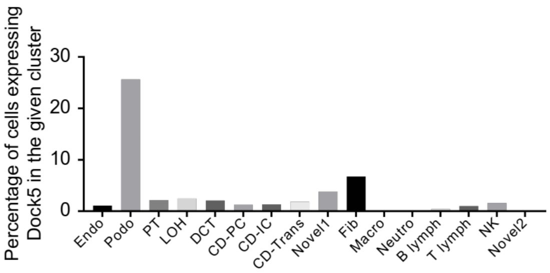

Figure S1. Dock5 shows a podocyte predominant expression pattern in line with published single-cell RNA sequencing datasets.

According to previous published single-cell RNA sequencing datasets [20], in mouse healthy kidney tissue, Dock5 showed a highest expression in podocytes in healthy mouse kidney tissue.

Endo, containing endothelial, vascular, and descending loop of Henle; Podo, podocyte; PT, proximal tubule; LOH, ascending loop of Henle; DCT, distal convoluted tubule; CD-PC, collecting duct principal cell; CD-IC, collecting duct intercalated cell; CD-Trans, collecting duct transitional cell; Fib, fibroblast; Macro, macrophage; Neutro, neutrophil; lymph, lymphocyte; NK, natural killer cell.

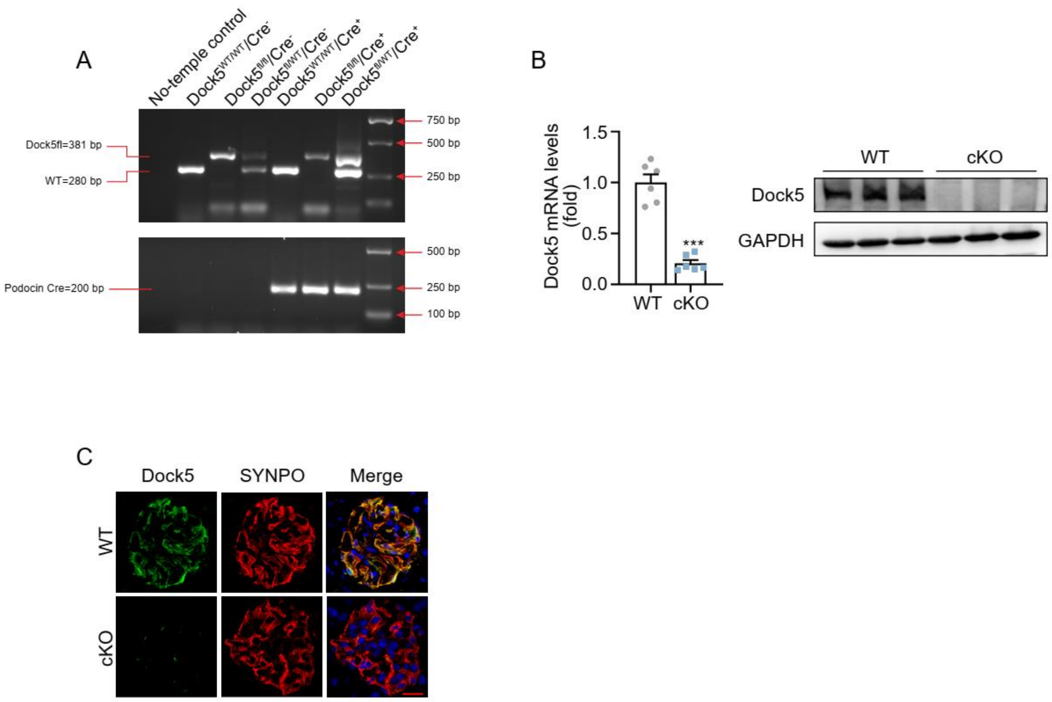

Figure S2. Dock5 expression is specifically deleted from podocytes.

(A-C) Podocyte-specific Dock5 knockout mice (Dock5$^{fl/fl}$-Cre^{+}) was generated by breeding Dock5 conditional allele (Dock5$^{fl/fl}$) with podocin-Cre mice. The genotypes were confirmed by tail genotyping (A), and podocyte deletion of Dock5 was confirmed by qRT-PCR and western blot analysis in isolated glomeruli (B) and by co-immunofluorescence staining for Dock5 (green) and synaptopodin (SYNPO, red) (C). Data are mean ± SEM. Scale bar: 20μm in C. n = 6 mice per group for A-C. Statistical analysis was performed using *t*-test for B. ****P* < 0.001.

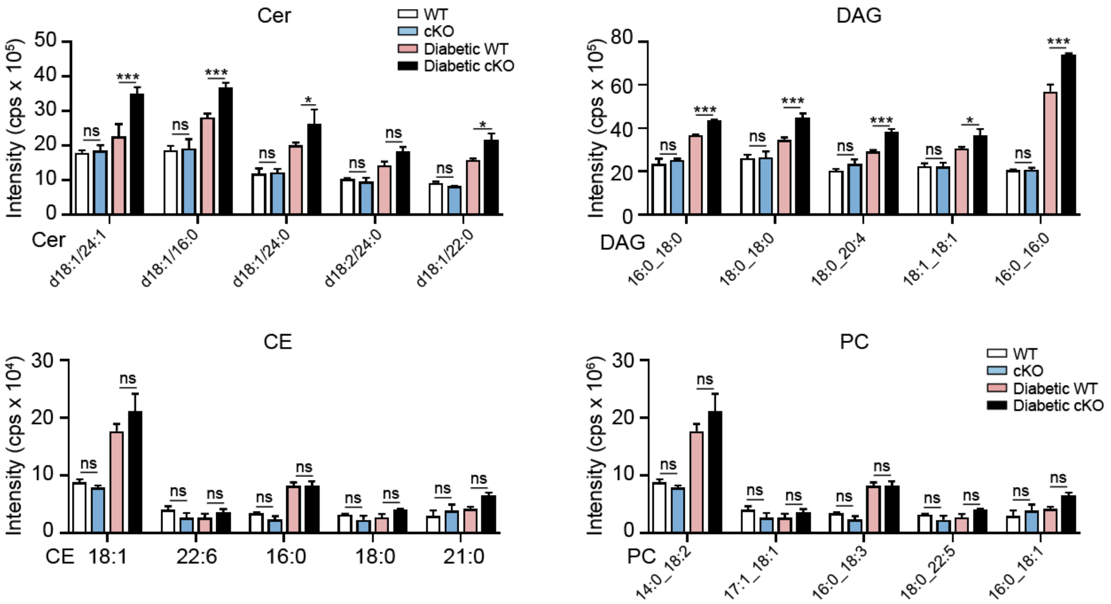

Figure S3. Dock5 deletion promotes toxic lipid accumulation, but does not influence cholesterol ester and phosphatidylcholines accumulation.

Lipidomics of podocytes isolated from indicated groups were assessed by UPLC-MS/MS analysis. Ion chromatograms and quantifications of indicated lipid specie were shown. Cer: Ceramides, DAG: diacylglycerols, CE: cholesterol ester and PC: phosphatidylcholines. Data are mean ± SEM. n = 3. Statistical analysis was performed using *t*-test. **P* < 0.05, ****P* < 0.001, ns, not significant.

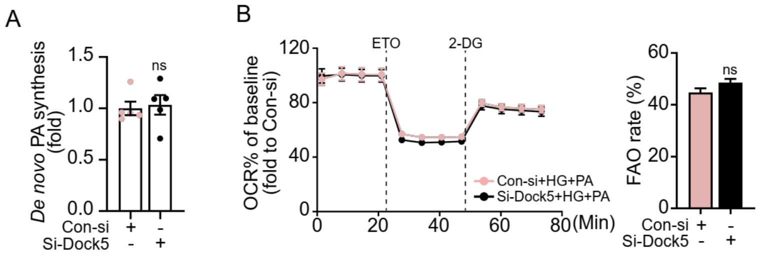

Figure S4. Dock5 has no effects on fatty acid de novo synthesis and oxidation.

(A) *De novo* lipogenesis was evaluated by ^{13}C-labaled glucose in podocyte treated by si-Dock5 under HG and PA treatment. (B) Relative oxygen consumption rate (OCR) of podocytes transduced with si-Dock5 under HG and PA treatment was measured by Seahorse experiment. Fatty acid oxidation (FAO) was calculated by etomoxir (ETO)-inhibitable OCR, and the FAO rate was quantified. Data are mean ± SEM. n = 5 for A, n = 3 for B. Statistical analysis was performed using (multiple) *t*-test. ns, not significant.

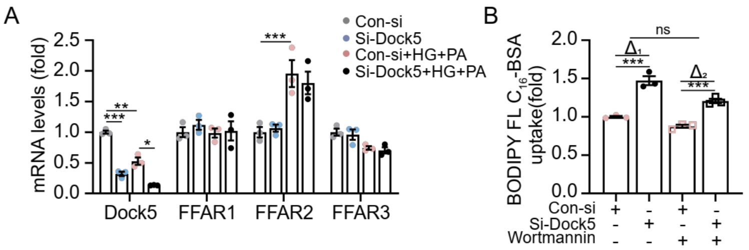

Figure S5. Dock5 deficiency induced fatty acid uptake is endocytosis-independent. (A) mRNA expression of indicated gene were assessed in podocytes transfected by Dock5 specific siRNA (si-Dock5) together with or without HG+PA. (B) BODIPY FL C_{16} evaluated PA uptake was conducted in podocytes transfected by si-Dock5 with or without macropinocytosis inhibitor wortmannin under HG and PA condition. Data are mean ± SEM. n = 3 for A and B. Statistical analysis was performed using one-way ANOVA test for A, *t*-test for B.**P* < 0.05, ***P* < 0.01, ****P* < 0.001, ns, not significant.

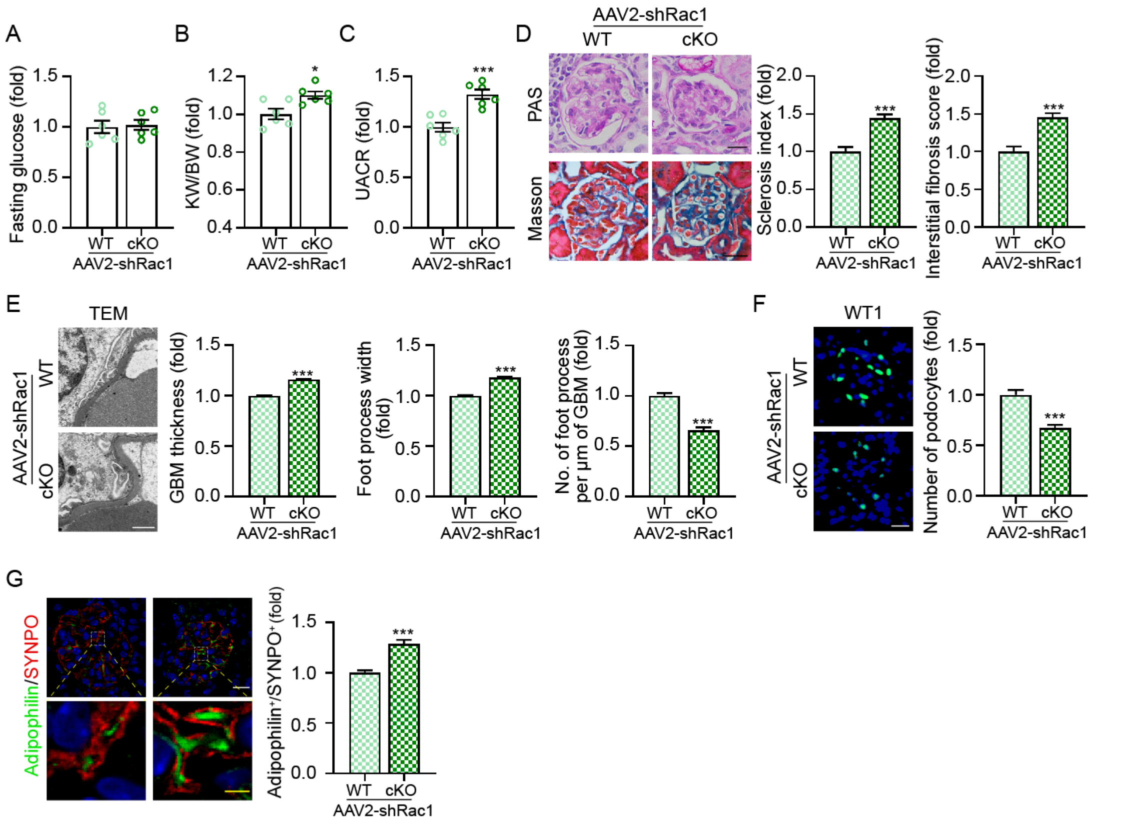

Figure S6. Dock5 deficiency exacerbates podocyte injury and lipotoxicity may independent of Rac1.

(A-D) Dock5 cKO and its wildtype (WT) littermates were induced diabetes by HFD feeding combined with STZ injection followed by AAV2-shRac1 injection. Fasting glucose levels (A), kidney weight-to-body weight ratio (KW/BW, B), urine albumin-to-creatinine ratio (UACR, C), and histological changes of glomeruli (assessed by Periodic acid–Schiff (PAS) and Masson's trichrome (Masson) staining) (D) were evaluated. (E) Representative TEM images and quantifications of GBM thickness, foot process width, and the number of foot processes in mice from indicated groups. (F) Representative images of WT1 staining in specific mouse groups were shown, and their quantifications were listed. (G) Representative IF images showing lipid accumulation in podocytes (co-staining adipophilin (green) with SYNPO (red)), and their quantifications are shown below. Data are mean ± SEM. Scale bar: 20μm in D and F, 1μm in E, 4 and 20μm in G (yellow bar represents 4μm). n = 6 mice per group for A-G, n = 10 images per mouse for E. Statistical analysis was performed using *t*-test.*$P < 0.05$, ***$P < 0.001$.

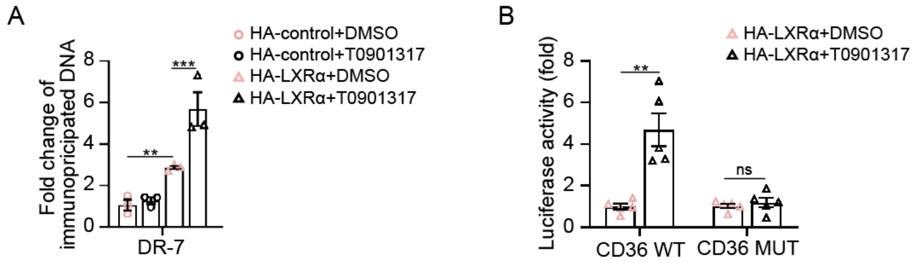

Figure S7. LXRα activates CD36 gene transcription via binding to its promoter region.

(A) HA-LXR or HA vector control was transfected into mouse podocytes, and then treated with DMSO or T0901317 (LXRα agonist) for 24 hours before ChIP assay using an anti-HA antibody. (B) Luciferase reporter assays to show that LXRα activated the CD36 promoter (CD36 WT), but not their DR-7 mutant variants (CD36 MUT), in the presence of T0901317. Data are mean ± SEM. n = 3 for A, n = 5 for B. Statistical analysis was performed using one-way ANOVA test for A, *t*-test for B.***P* < 0.01, ****P* < 0.001, ns, not significant.

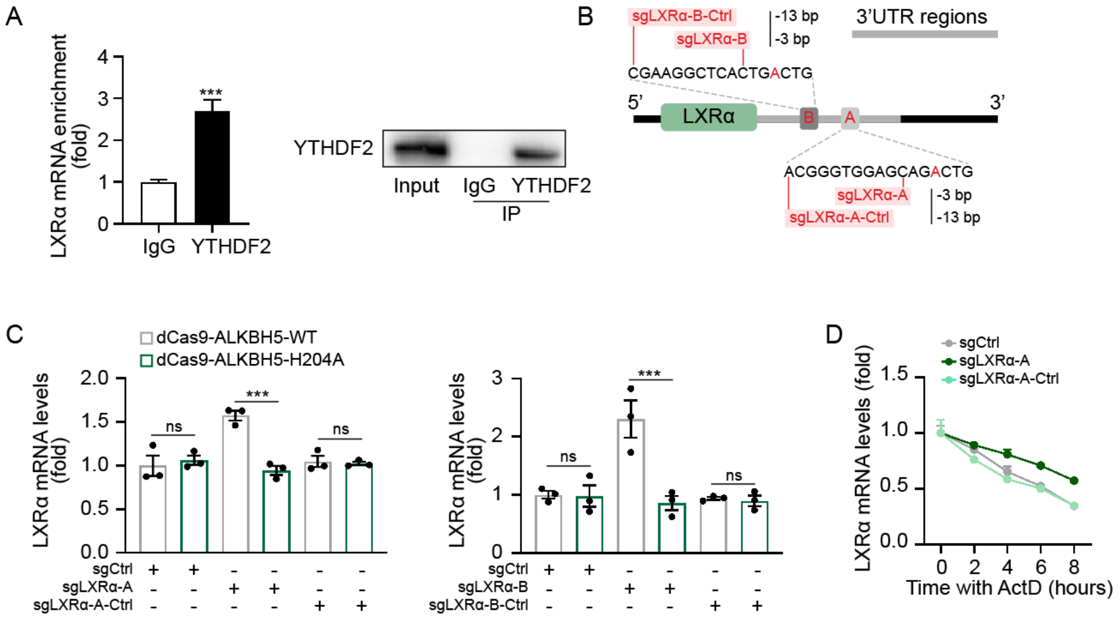

Figure S8. The m^{6}A modification per se expedites the degradation of LXRα mRNA.
(A) Podocyte lysates were incubated with YTHDF2 antibody, then the RNA was extracted for RIP analysis. (B) Schematic illustration showing the effective (sgLXRα-A or sgLXRα-B) and noneffective (10 bp away from the former, sgLXRα-A-Ctrl or sgLXRα-B-Ctrl) sgRNAs targeting LXRα transcript. (C and D) HG and PA-treated podocytes were transfected with dCas9-ALKBH5-WT or the incompetent demethylase (dCas9-ALKBH5-H204A) together with indicated sgRNA. mRNA expression (C) and mRNA half-life (D) of LXRα were assessed. ActD were added to inhibit the transcription. Data are mean ± SEM. n = 3 for A, C and D. Statistical analysis was performed using *t*-test for A and C. $***P < 0.001$, ns, not significant.

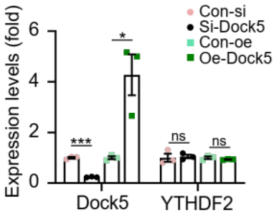

Figure S9. Dock5 has no impact on YTHDF2 mRNA expression.

HG and PA-treated podocytes were transfected with Dock5 siRNA or its overexpression plasmids, mRNA expression of Dock5 and YTHDF2 were assessed by qRT-PCR. Data are mean ± SEM. n = 3. Statistical analysis was performed using *t*-test. **P* < 0.05, ****P* < 0.001, ns, not significant.

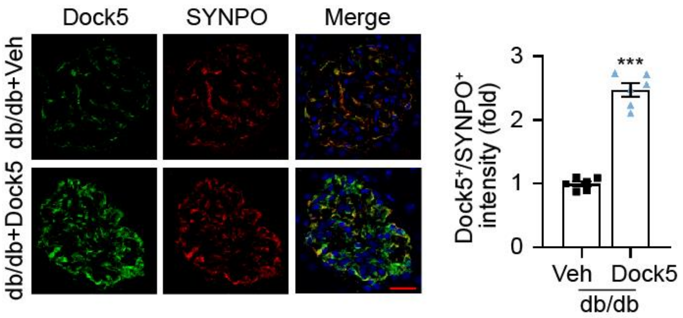

Figure S10. Dock5 expression is rescued in podocyte from db/db diabetic mice by Ad-Dock5 treatment.

Co-immunostaining and its quantifications of Dock5 and SYNPO in kidney tissue from Ad-Dock5 treated db/db DKD mice. Scale bar: 20μm. n = 6 mice per group. Data are mean ± SEM. Statistical analysis was performed using *t*-test. ***$P < 0.001$.
