# Supplementary material for: Dock5 Deficiency Promotes Proteinuric Kidney Diseases via Modulating Podocyte Lipid Metabolism
Source: Adv Sci (Weinh). 2023 Dec 31;11(11):2306365. doi: 10.1002/advs.202306365 (PMC10953540; doi:10.1002/advs.202306365)
Supplement: Supplementary file 4 — Supplemental Table 3 [file ADVS-11-2306365-s003.pdf]

ADVANCED SCIENCE
Open Access

Supporting Information

for *Adv. Sci.*, DOI 10.1002/advs.202306365

Dock5 Deficiency Promotes Proteinuric Kidney Diseases via Modulating Podocyte Lipid Metabolism

Hua Qu, Xiufei Liu, Jiaran Zhu, Xin Xiong, Lu Li, Qingshan He, Yuren Wang, Guojun Yang, Linlin Zhang, Qingwu Yang, Gang Luo, Yi Zheng* and Hongting Zheng**

Table S3: Clinical and laboratory characteristics of all participants.

	Ctrl	DKD
**Sex (M/F)**	7 (4/3)	6 (4/2)
**Age (year)**	51.57 ± 8.88	55.83 ± 5.98
**BMI (kg/m^2)**	22.91 ± 2.56	24.78 ± 2.21
**SBP (mm Hg)**	118.00 ± 7.30	128.00 ± 9.25
**DBP (mm Hg)**	75.29 ± 8.26	84.83 ± 3.87[a]
**FPG (mmol/L)**	4.77 ± 0.52	8.55 ± 1.40 [b]
**HbA$_{1C}$ (%)**	5.49 ± 0.15	8.58 ± 1.03 [b]
**TC (mmol/L)**	4.45 ± 0.47	5.31 ± 0.95
**TG (mmol/L)**	1.06 ± 0.09	2.64 ± 2.08
**HDL-C (mmol/L)**	1.16 ± 0.17	0.94 ± 0.27
**LDL-C (mmol/L)**	2.21 ± 0.45	2.14 ± 0.97
**BUN (mmol/L)**	4.69 ± 1.14	8.87 ± 3.89[a]
**Cr (μmol/L)**	56.79 ± 11.40	154.90 ± 22.85[b]
**eGFR (ml/min/ 1.73m^2)**	113.71 ± 8.67	39.50 ± 5.01[b]
**UACR (mg/g)**	0.83 ± 1.02	390.75 ± 58.75[b]

Data are presented as means ± SD. Ctrl, control subjects; DKD, diabetic kidney disease; BMI, body mass index; SBP, systolic blood pressure; DBP, diastolic blood pressure; FPG, fasting plasma glucose; HbA1c, glycosylated hemoglobin; TC, total cholesterol; TG, triglyceride; HDL-c, high-density lipoprotein-cholesterol; LDL-c, low-density lipoprotein-cholesterol; BUN, urea; Cr, Serum creatinine; eGFR, estimated glomerular filtration rate; UACR, urea albumin to creatinine ratio.

[a]$P < 0.05$ compared with Ctrl ; [b]$P < 0.01$ compared with Ctrl.

Table S4. List of primers for ChIP-qPCR.

**Primers**		
**CD36-DR-7**	ChIP-F	GGGTTTACAAGATGGTGTCATCAC
	ChIP-R	TTAGCCTCATGAGGTTTCCCAGG
**CD36-Control**	ChIP-F	TGAAGGCTTCTCACATATTGTGG
	ChIP-R	TGTCTATGGGGAAGGATTATGTC
**GAPDH**	ChIP-F	CACGTCCCAACTCTCCACCCTG
	ChIP-R	GGTTGAATTGGAGGAGGCTCAGAG

Table S5. List of primers.

**Primers**

**Dock5**	Forward	GAGGGCCTAGGCAGTCTCTT
	Reverse	ACTGGGTCAGCAACCTCAAG
**Fn1**	Forward	GCAGTGACCACCATTCCTG
	Reverse	GGTAGCCAGTGAGCTGAACAC
**FFAR1**	Forward	CCTGAGCCACAAACGGA
	Reverse	AGCCACATTGGAGGCATT
**FFAR2**	Forward	TGCCCAGAGAAGCATAGC
	Reverse	CAGCATCACCAGCCAGA
**FFAR3**	Forward	TGGCATCGGCTCACTGTA
	Reverse	TCCCCTGGCTGTAGGTTG
**CD36**	Forward	CCTCCAGAATCCAGACAACC
	Reverse	CACAGGCTTTCCTTCTTTGC
**FABP4**	Forward	AATCACCGCAGACGACA
	Reverse	ACATTCCACCACCAGCTT
**CPT1a**	Forward	TGTGGCTTGCTGTATTTGA
	Reverse	TGACTGGGTGGGATTAGAA
**LXRa**	Forward	TCTCAAGAGCCACGTACCAAGAC
	Reverse	GTTGTACCTCCGTGACGTCTCC
**Pre-LXRa**	Forward	GTTGGAGTCAGCAGAGCCTACAG
	Reverse	TGTGACGTTAGAAAGCAAAGAGCC
**YTHDF2**	Forward	GGATGGCAGCACTGAAA
	Reverse	CTGGTTTTGGAGGAGCAA
**TGFB3**	Forward	GGACTTCGGCCACATCAAGA
	Reverse	ATAGGGGACGTGGGTCATCA
**GM50207**	Forward	CAATGCCCCAGTGGACTAGC
	Reverse	TGCACTTCTTCCACGATGCT
**GM35853**	Forward	TGCCTCACGTCTTCATCGAG
	Reverse	GCCTTCTCCAGTACAGCTCC
**PLPP3**	Forward	GTGTTCTTCGTGTCCGACCT
	Reverse	GTCGATGATGTCCACGGGAG
**INMT**	Forward	CTCTGCGCTTCCAGCACTAT
	Reverse	GCAGTAAGCCTCCGAGTAGC
**CAVIN2**	Forward	ATGACGAATTGCCCCGTGAT
	Reverse	TGGTTGGGTGTGAGCGATTT
**ATP2B4**	Forward	AGTGAAGGGCTCCGGACTAT

	Reverse	ATCCTCAATGCCCACCACTG
**GAPDH**	Forward	TGAACGGGAAGCTCACTG
	Reverse	TCCACCACCCTGTTGCTG

Table S6. sgRNA oligos and PAMers used in CRISPR-based m^{6}A-editing tools.

Name	5'-3' Sequences
**sgLXRα-A**	CACCGCTGCTTCCCACGGGTGGAGC
**sgLXRα-A-Ctrl**	CACCGGGCTCACTGACTGCTTCCCA
**sgLXRα-B**	CACCGGCCACATGGCGAAGGCTCAC
**sgLXRα-B-Ctrl**	CACCGCTTTGTGTTGGCCACATGGC
**sgControl**	CACCGGAATAGCTCAGAGGCCGAGG
**LXRα-A-PAMer**	mGdGmAdAmGdCmAdGmUdGdGmGdUmGdAmGdCmCdTmUdCmGdCmCdAmUdG
**LXRα-A-Ctrl -PAMer**	mAdGmUdGmAdGmCdCmUdGdGmGdCmCdAmUdGmUdGmGdCmCdAmAdCmAdC
**LXRα-B-PAMer**	mCdAmUdGmUdGmGdCmUdGdGmCdAmCdAmAdAmGdGmAdCmAdCmGdGmUdG
**LXRα-B-Ctrl -PAMer**	mAdCmAdCmAdAmAdGmUdGdGmAdCmGdGmUdGmAdAmAdCmAdGmUdCmAdC
